# Linzagolix, with and without add-back therapy, in women with endometriosis-associated pain: results from EDELWEISS 6, a double-blind randomized extension and withdrawal study

**DOI:** 10.1093/hropen/hoag030

**Published:** 2026-04-08

**Authors:** Jacques Donnez, Christian M Becker, Felice Petraglia, Andrew W Horne, Sven Becker, Stefan P Renner, Francisco Carmona Herrera, Hugh S Taylor, Maciej Paszkowski, Elke Bestel, Satoshi Hori, Marie-Madeleine Dolmans

**Affiliations:** Department of Gynecology, Université Catholique de Louvain, Brussels, Belgium; Department of Gynecology, Société de Recherche pour l‘Infertilité (SRI), Brussels, Belgium; Endometriosis CaRe Centre, Nuffield Department of Obstetrics and Gynaecology, University of Oxford, Oxford, UK; Obstetrics and Gynecology Unit, Department of Clinical Experimental and Biomedical Sciences, University of Florence, Florence, Italy; EXPPECT Edinburgh Centre for Reproductive Health, University of Edinburgh, Edinburgh, UK; Department of Gynecology and Obstetrics, Frankfurt Goethe University, Frankfurt, Germany; Department of Gynecology and Obstetrics, Hospital Böblingen, Klinikverbund-Suedwest, Sindelfingen, Germany; Endometriosis Unit, Hospital Clinic of Barcelona, University of Barcelona, Barcelona, Spain; Department of Obstetrics, Gynecology and Reproductive Sciences, Yale School of Medicine, New Haven, CT, USA; Third Chair and Department of Gynecology, Medical University of Lublin, Lublin, Poland; Department of Medical Affairs, Theramex, London, UK; Department of Medical Affairs, Theramex, London, UK; Gynecology Research Laboratory, Department of Gynecology, Institut de Recherche Expérimentale et Clinique, Université Catholique de Louvain, Brussels, Belgium; Gynecology Department, Cliniques Universitaires St-Luc, Brussels, Belgium

**Keywords:** endometriosis, pain, dysmenorrhea, non-menstrual pelvic pain, quality of life, linzagolix

## Abstract

**STUDY QUESTION:**

Was the efficacy of linzagolix, observed at 6 months in women with endometriosis-associated pain, maintained when administered for an additional 6 months?

**SUMMARY ANSWER:**

Efficacy from this extension study complements the results of the main trial, by showing that linzagolix is effective at reducing endometriosis-associated pain for a majority of women.

**WHAT IS KNOWN ALREADY:**

In the pivotal EDELWEISS 3 randomized controlled trial, the efficacy and safety of 24 weeks of once-daily oral linzagolix (a GnRH antagonist) were reported in women with endometriosis-related pain. Risks of bone mineral density (BMD) loss and vasomotor symptoms were minimized in both groups.

**STUDY DESIGN, SIZE, DURATION:**

EDELWEISS 3 was a prospective, randomized, double-blind study. Subjects completing the 6-month treatment period in EDELWEISS 3 were invited to participate in the present extension study: EDELWEISS 6. Only subjects who had completed the full 6-month treatment course in the main study and who met the inclusion criteria were eligible for entry into the extension study. Among 486 patients who completed the 6-month study, 353 participated in the extension study from March 2020 to February 2023. The current extension study started at Month 6 of the main EDELWEISS 3 phase-3 trial. Upon the end of treatment in the extension study (6-month treatment period from Month 6 to Month 12), subjects entered a post-treatment follow-up period of 6 months. The current report describes outcomes from the extension treatment period (up to 12 months) and subsequent follow-up of 6 months with no investigational medicinal product. The co-primary endpoints were the reduction in dysmenorrhea and non-menstrual pelvic pain at Month 12.

**PARTICIPANTS/MATERIALS, SETTING, METHODS:**

Premenopausal women with moderate-to-severe endometriosis who previously completed the full 6-month treatment period in the main study and met the inclusion criteria were eligible for the extension study. All subjects (n = 353), whether given placebo or not in the main study, received either 75 mg linzagolix alone or 200 mg combined with hormonal add-back therapy (ABT) once daily for the additional 6 months, i.e. those who were given a placebo during the main study were randomized to either group and those who had received active treatment continued with the same treatment (placebo/75 mg linzagolix [n = 57], placebo/200 mg linzagolix plus ABT [n = 57], 75 mg linzagolix [n = 118], and 200 mg linzagolix plus ABT [n = 121]). Pain was measured daily on a verbal rating scale and recorded in an electronic diary. The trial was performed in clinics and university hospitals in the USA and Europe.

**MAIN RESULTS AND THE ROLE OF CHANCE:**

By Month 12, of the 353 women who were treated and analyzed, the proportion of subjects with a significant reduction in dysmenorrhea and stable or decreased use of analgesics was 55.9% in the 75 mg linzagolix group and 91.0% in the 200 mg+ABT linzagolix group. The proportion of subjects with a significant reduction in non-menstrual pelvic pain and stable or decreased use of analgesics was 59.5% in the 75 mg linzagolix group and 67.6% in the 200 mg+ABT linzagolix group. A steady reduction in mean daily dyschezia, dyspareunia, and overall pelvic pain scores was observed in both linzagolix groups from baseline to Month 12, with a more marked reduction in the 200 mg+ABT linzagolix group. Quality of life was assessed using the EHP-30 questionnaire. At Month 12, clear improvements (total score reductions) were observed in both linzagolix groups, with greater improvements in five out of five dimensions in the 200 mg+ABT linzagolix group. Changes in body mass index were minimal, with no clinically meaningful modifications from Month 6 to Month 12. Adverse events including hot flushes were encountered in fewer than 1% of subjects. During the off-treatment follow-up period of 6 months, some of the improvements were maintained for several months.

**LIMITATIONS, REASONS FOR CAUTION:**

Efficacy was originally compared between the linzagolix and placebo groups from the main study, however, it would have been useful to have results from comparative studies with estro-progestogens or progestogens, to ascertain whether a GnRH antagonist has significant benefits over traditional first-line medications.

**WIDER IMPLICATIONS OF THE FINDINGS:**

Linzagolix, when administered orally once daily at a dose of 200 mg in combination with ABT, offers an additional option for effective, safe, and well-tolerated medical treatments for endometriosis that can be proposed in cases of estro-progestogen or progestogen inefficacy, as it can be used for a longer term, improving quality of life and reducing the need for analgesics and opioids. The risks of bone mineral loss and vasomotor symptoms were minimized due to the ABT. The 75 mg dose alone could be suitable for chronic treatment of endometriosis-associated pain without the need for concomitant hormonal ABT and may offer an option for some women, although further research is needed to confirm this finding.

**STUDY FUNDING, COMPETING INTEREST(S):**

Funding for the EDELWEISS 6 study was provided by Geneva, Switzerland. Analysis of data was partially supported by ObsEva (Geneva, Switzerland), Theramex (London, UK), and Kissei (Japan). Grant 5/4/150/5 was awarded to M.-M.D. by the Fonds National de la Recherche Scientifique (FNRS). J.D. was a member of the scientific advisory board of ObsEva and Preglem until 2023 and reports consulting fees from ObsEva, Gedeon Richter, and Theramex. C.M.B. declares consulting fees (paid to University of Oxford) from Myovant for an advisory board and from Theramex; payment or honoraria (paid to University of Oxford) for talks at different meetings from [Theramex and Gedeon Richter]; support for attending meetings and/or travel (paid to University of Oxford) from [Gedeon Richter]; being a member of the independent data monitoring board for the EDELWEIS 3 trial and member of the advisory board for Myovant for Spirit 1 and 2 trials (funds for both were paid to University of Oxford); being Chair of the ESHRE Endometriosis Guideline Committee (unpaid) and a member of the Medical Advisory Board Endometriosis UK (unpaid). F.P. has received fees and honoraria for lectures from Theramex. A.W.H. declares consultancy fees (paid to institution) from Roche Diagnostics, Gesynta, and Theramex; lecture fees from Gedeon Richter and Theramex; grant funding from the EU, UKRI, NIHR, CSO, Wellbeing of Women and Roche Diagnostics; UK Patent 2217921-2; participation on a Data Safety Monitoring Board for PANDA DMC; and roles as Trustee and Medical Advisor to Endometriosis UK, and Specialty Advisor to the Scottish Government’s Chief Medical Officer for Obstetrics and Gynaecology. S.B. has received consulting fees and honoraria for lectures from Theramex. S.P.R. reports consulting fees and honoraria for lectures from Gedeon Richter and Theramex. F.C.H. reports consulting fees and honoraria for lectures, presentations, or educational events from Theramex and Gedeon Richter; and honoraria for participation in a data safety monitoring board for Organon. HT has received grants from Abbvie, reports consulting fees from ObsEva and Gedeon Richter and stock options from DotLab; declares a patent on endometriosis biomarkers, owned by Yale University (US10 982 282). M.P. was a principal investigator in the ObsEva-sponsored EDELWEISS trials and has received fees and honoraria from Theramex. E.B. and S.H. are employees of Theramex. M.-M.D. has received fees for lectures from Gedeon Richter and Theramex.

**TRIAL REGISTRATION NUMBER:**

NCT04335591 (ClinicalTrials.gov).

WHAT DOES THIS MEAN FOR PATIENTS? Endometriosis affects almost 10% of women during their reproductive years and is associated with pain symptoms like dysmenorrhea (pain during menstruation), chronic pelvic pain, and dyspareunia (pain during sexual intercourse), with serious impacts on quality of life. Surgical excision can be effective for relief of pain, but lesions may recur relatively frequently, requiring medical pain management or more surgery. Medical hormonal therapy, like combined oral contraceptives or progestogens, is effective for about two-thirds of women with pain related to endometriosis. However, for the remaining women, there is a need for novel medical treatments, such as oral use of antagonists to gonadotrophin-releasing hormone (GnRH). The present study extended a previous trial investigating whether linzagolix, an oral GnRH antagonist, taken once a day, can continue to alleviate symptoms associated with endometriosis. Indeed, the present report shows that 200 mg linzagolix, when taken with add-back hormonal therapy (i.e. low doses of both estrogens and progestogens), continued to relieve endometriosis-associated pain for a further 6 months, with minimal side effects such as bone-mineral loss. Thus, this therapy may be proposed for women when oral contraceptives or progestogen are ineffective.

## Introduction

Endometriosis is a chronic inflammatory disorder which affects almost 10% of women during their reproductive years. Endometriosis-associated pain symptoms, including dysmenorrhea, chronic non-menstrual pelvic pain (NMPP), dyspareunia, and dyschezia (Nisolle and Donnez 1997; Giudice and Kao, 2004; Parazzini *et al.*, 2020; Zondervan *et al.*, 2020; Donnez and Dolmans, 2021a,b; Taylor *et al.*, 2021; Bonavina and Taylor, 2022; Donnez and Cacciottola, 2022; Horne and Missmer, 2022) can seriously impact quality of life (QoL) for the affected women. As it often results in loss of productivity at work (Pluchino *et al.*, 2016; Barbara *et al.*, 2021), endometriosis constitutes a substantial economic burden, as well as a considerable financial outlay for society and health insurance systems (Gao *et al.*, 2006; Simoens *et al.*, 2012; Soliman *et al.*, 2016, 2017).

Existing therapies for endometriosis are either medical or surgical (Donnez and Dolmans, 2021a,b). Surgical removal of endometriotic lesions may be effective for relief of pain short-term (Donnez *et al.*, 2002), but lesions recur relatively frequently and therefore require repeated surgery or courses of medical pain management (Bozdag, 2015; Donnez, 2021; Capezzuoli *et al.*, 2021). Identifying the most appropriate medical therapy is challenging. First-line treatments for endometriosis, such as combined oral contraceptives (COCs) or progestogens, work by preventing ovulation and reducing menstrual bleeding (Vercellini *et al.*2018a,b). However, they are only effective in dealing with pain related to endometriosis for about two-thirds of women (Vercellini *et al.*, 2016, 2018a,b; Donnez and Dolmans, 2021a,b). The failure rate with progestogens is largely attributed to progesterone resistance in lesions (Bulun *et al.*, 2019, 2023; Yilmaz and Bulun, 2019; Donnez and Dolmans, 2021a; Taylor *et al.*, 2021; Cacciottola *et al.*, 2021). In addition, many patients have significant side effects, with about one-third developing depression (Cevik and Taylor, 2025). Second-line treatments, such as injectable depot formulations of GnRH agonists, are generally only offered if COCs or progestogens fail, as they are associated with the early onset of menopausal symptoms (Barbara *et al.*, 2021; Donnez and Dolmans, 2021a,b; Becker *et al.*, 2022) like bone mineral density (BMD) loss and hot flushes, and therefore require hormonal add-back therapy (ABT) if used for more than 6 months. Thus, novel medical treatments for endometriosis are required.

According to the estrogen threshold hypothesis (Barbieri, 1992), regulating estrogen levels to minimize menopausal symptoms, while preserving effectiveness in terms of reducing endometriosis-related symptoms, may be one treatment approach (Donnez *et al.*, 2017). Oral GnRH antagonists have therefore emerged as an alternative as they offer dose-dependent regulation of estradiol (E2) levels (Donnez *et al.*, 2017). In two similar double-blind, randomized, phase-3 trials, elagolix, a non-peptide oral GnRH antagonist administered at doses of 150 mg once daily or 200 mg twice daily, was demonstrated to be effective at treating endometriosis-associated pain (Taylor *et al.*, 2017). However, elagolix has a short half-life of 4–6 h, so twice-daily administration of the higher dose is required to achieve enough suppression of endogenous estrogen production (Ng *et al.*, 2017). Additionally, relugolix combination therapy (40 mg relugolix plus hormonal ABT) was reported to offer significant relief of endometriosis-associated pain, and this effect was sustained for up to 2 years (Giudice *et al.*, 2022; Becker *et al.*, 2024).

Linzagolix is a non-peptide oral GnRH antagonist which, like elagolix and relugolix, acts by binding to and blocking the GnRH receptor in the pituitary gland, resulting in a dose-dependent decrease in the production of LH and FSH (Donnez *et al.*, 2017, 2020, 2022, 2023). Linzagolix can be given once a day (Donnez *et al.*, 2020, 2022) and at suitable doses, to maintain estradiol concentrations within the desired range of 20–60 pg/ml. This may be optimal for alleviating pain associated with endometriosis, while minimizing loss of BMD and other undesirable effects linked to complete estrogen suppression (Donnez *et al.*, 2017, 2020). However, at high doses, linzagolix reduces estradiol levels to below 20 pg/ml, which is regarded as full suppression (Donnez *et al.*, 2017, 2020).

In a recent paper on the pivotal EDELWEISS 3 randomized controlled trial (RCT) (Donnez *et al.*, 2024), we reported the efficacy and safety of 24 weeks of once-daily oral linzagolix (200 mg+ABT (1 mg estradiol and 0.5 mg of norethisterone acetate) or 75 mg alone) in women with endometriosis-related pain. QoL was found to be improved. Risks of BMD loss and vasomotor symptoms were minimized in the group on 200 mg+ABT, due to the additional effect of ABT, and in the 75 mg group, due to the only partial suppression of E2. We concluded in the paper that the 200 mg dose + ABT could be proposed in cases of estro-progestogen or progestogen inefficacy and used for a longer term, to improve quality of life while minimizing the risks of BMD loss. Based on this pivotal trial results, 200 mg linzagolix with ABT was recently authorized by the European Commission for the symptomatic treatment of endometriosis in women with a history of previous ineffective medical or surgical treatment. The 75 mg dose alone could also be suitable without the need for concomitant hormonal ABT; however, further research was needed to confirm this.

Here, we report on the long-term (12 months) efficacy and safety results of 75 mg linzagolix alone, and 200 mg linzagolix with ABT, in the treatment of endometriosis-associated pain from the EDELWEISS 6 extension study, as well as an additional follow-up period (6 months) after treatment cessation.

## Materials and methods

### Study design

This was a prospective, randomized, double-blind study. Subjects completing the 6-month treatment period in EDELWEISS 3 were invited to participate in the present extension study. Only subjects who had completed the full 6-month treatment course in the main study and met the inclusion criteria were eligible for entry into the extension study. The Month 6 visit of the main study was a juncture for subjects to decide whether to end treatment or opt for a 6-month extension. They were required to sign a specific informed consent form in order to continue.

The primary objective of the extension study was to assess maintenance of linzagolix efficacy when administered orally once daily for an additional 6 months (up to 12 months of treatment in total). Women with surgically confirmed endometriosis, who had already completed 6 months of treatment for management of moderate-to-severe endometriosis-associated pain, were recruited.

The trial was granted approval from the applicable ethics committee or institutional review board of each participating center and was conducted in line with International Conference on Harmonization guidelines, relevant regulations and appropriate ethical principles derived from the Declaration of Helsinki. It was registered on ClinicalTrials.gov with the identifier NCT04335591. All prospective study participants provided informed written consent before any screening activities. The sponsor, ObsEva S.A., was responsible for designing the trial. To maintain consistency in such large multinational and geographically diverse trials, the sponsor engaged a global contract research organization (Fortrea Inc., Princeton, NJ, USA) to identify investigational sites, set up countries and locations, oversaw data collection, and conducted site monitoring, ensuring that the same procedures and standards were used throughout. Data were analyzed by a different contract research organization (Cytel Inc., Cambridge, MA, USA), which similarly used identical rules for all countries/sites involved.

### Patients

As previously reported, participants enrolling in the main EDELWEISS 3 study were premenopausal women aged 18–49 years with moderate-to-severe endometriosis symptoms (with moderate-to-severe dysmenorrhea and NMPP over the course of two screening menstrual cycles). Patients had to have completed the 6-month treatment course in the main study and be willing and able to continue to comply with protocol requirements for the duration of the extension study. They also had to agree to continue using only the analgesic rescue medication permitted by the protocol during the treatment and follow-up periods. Among 486 patients who completed the 6-month study, 353 participated in the extension study. Indeed, of the 356 subjects who entered the extension study, three ceased treatment prematurely based on discontinuation criteria at Month 6 and were thus not included in the treatment extension analysis set. After cessation of therapy, patients were followed for another 6-month period (Extension follow-up, EXFU).

### Randomization

Prior to the start of the main study, a randomization list had been generated by computer randomization using Statistical Analysis System software, version 9.4 (SAS Institute Inc., Cary, NC, USA). They were sent to the assigned clinical packaging organization for labeling, and to a fully integrated interactive web response system (IWRS) for kit numbers. Subjects who had been taking a placebo were randomized to one of two treatment groups at a 1:1 ratio according to the randomization schedule applied in the main study. Subjects who had been on active treatment in the main study simply continued with the same treatment they had been taking for the first 6 months.

### Efficacy assessments

Similar to the main study, the subject’s assessment of dysmenorrhea and non-menstrual pelvic pain was collected daily via an eDiary using a verbal rating scale (VRS). In EDELWEISS 3, dysmenorrhea and non-menstrual pelvic pain were calculated by averaging the daily e-diary answers to the endometriosis-related pelvic pain questionnaire over the corresponding 28 calendar days prior to the assessment point. The subject selected the category that best described her endometriosis-associated pelvic pain in the last 24 h: 0—No pain; 1—Mild pain but I was easily able to do the things I usually do; 2—Moderate pain: I had some difficulty doing the things I usually do; and 3 –Severe pain: I had great difficulty doing the things I usually do.

Responses of ‘No pain’, ‘Mild pain’, ‘Moderate pain’, and ‘Severe pain’ were assigned a score of 0, 1, 2, and 3, respectively. Dysmenorrhea was counted days with uterine bleeding, defined as those on which the patient recorded any uterine bleeding or spotting in her e-diary; while non-menstrual pelvic pain was counted days with no uterine bleeding.

If a patient’s mean score for dysmenorrhea was undefined numerically because her daily e-diary indicated that she did not experience any uterine bleeding at all during the 28-day calendar period, her mean score for dysmenorrhea was set equal to zero (reflecting the absence of any dysmenorrhea during that time period). Similarly, if a subject’s diary reports showed that she only experienced days with uterine bleeding during the period, then the mean score for non-menstrual pelvic pain was set equal to zero.

Efficacy endpoints in EDELWEISS 6 were derived in the same way as in the main study. The same criteria for defining a subject as being a responder based on clinically meaningful reductions in dysmenorrhea and non-menstrual pelvic pain, along with stable or decreased use of analgesics for endometriosis-associated pain, were used, as in the main study.

In addition, the subject rated her worst endometriosis-associated pelvic pain and her dyschezia in the last 24 h on a 0–10 numerical rating scale (NRS), with 0 representing no pelvic pain and 10 representing the worst pain imaginable.

Dyspareunia was assessed with a VRS to rate how her endometriosis-associated pain interfered with sexual intercourse in the last 24 h with the following response options: 0—No pain during sexual intercourse; 1—I was able to tolerate the pain during sexual intercourse; 2—Intercourse was interrupted due to pain; or 3—I avoided sexual intercourse because of anticipation of pain. The patient was given an option to indicate no sexual activity for reasons other than her endometriosis; in such case, no score was allocated.

Further, the subject assessed daily function, uterine bleeding, analgesic use, having to miss school/work or cancel events, sleeping or lying down during the day, experiencing difficulty sleeping due to endometriosis-associated pain and perceived impression of severity (patient global impression of severity [PGIS]). Outcome rating scores were evaluated at each visit. Other efficacy assessments such as the endometriosis health profile (EHP-30), patient global impression of change (PGIC), and the EuroQoL 5-dimension 5-level (EQ-5D-5L) questionnaire were assessed at monthly on-site visits.

### Safety assessments

Safety assessments included all adverse events (AEs), investigating their severity and relationship to the study drug by physical examination and vital signs. BMD was appraised via dual-energy X-ray absorptiometry (DXA). Other safety evaluations were performed by transvaginal ultrasound (TVUS), endometrial biopsies, laboratory parameters (hematology, blood chemistry, hormone levels, and urine), electrocardiograms, and the Columbia-Suicide Severity Rating Scale (C-SSRS) questionnaire.

The following safety endpoints were planned: (i) change from baseline to each scheduled assessment of BMD measured by DXA of the lumbar spine (L1–L4), femoral neck, and total hip. (BMD, expressed as both absolute values and *Z*-scores, were summarized at each visit in terms of actual values and changes from baseline for each anatomic location. For absolute values only, percentage change from baseline and 95% confidence intervals for mean percentage change from baseline within each group were produced at each timepoint. Percent change from baseline in BMD was summarized as the number of subjects in predefined categories: no change or any increase; ≤3% decrease; >3% and ≤5% decrease; >5% and ≤7% decrease; >7% and ≤8% decrease; and >8% decrease.); (ii) incidence and severity of treatment-emergent adverse events (TEAEs); (iii) incidence and severity of hypoestrogenic TEAEs (hot flushes); (iv) time to the first post-treatment menses; (v) changes in clinical laboratory assessments (hematology, biochemistry, coagulation parameters, hormones, lipids, and urinalysis) from baseline to each scheduled assessment; and (vi) any pathological changes from baseline in the endometrium, as determined by histology from endometrial biopsies and endometrial thickness.

### Outcomes

The two co-primary efficacy endpoints were (i) a clinically meaningful reduction in dysmenorrhea and non-menstrual pelvic pain over the last 28 days of randomized treatment up to the Month 12 visit, and (ii) stable or decreased use of analgesics for endometriosis-related pain. For each of the co-primary endpoints, criteria used to define a subject as a responder over the previous 28 days of randomized treatment up to Month 12 included a reduction in dysmenorrhea of 1.10 or more on the VRS and in NMPP of 0.80 or more from baseline, as well as steady or diminished use of analgesics for endometriosis-associated pain (namely thresholds acquired from Month 3 meaningful change threshold analyses, as reported in Donnez *et al.*, 2024).

Secondary objectives included assessment of pain associated with sexual intercourse (dyspareunia) and defecation (dyschezia), difficulty doing daily activities, analgesic consumption, patient perception of severity, changes in uterine bleeding, and general QoL, as well as information gathered using questionnaires. Safety and tolerability objectives included evaluation of BMD, endometrial health, standard laboratory safety parameters, and adverse event (AE) frequency, including specific hypoestrogenic AEs.

### Statistical methods

Since this was an extension study, sample size was determined by the number of patients who were completing the main EDELWEISS 3 study and were eligible and willing to participate in the extension study. The main study baseline (Donnez *et al.*, 2024) was used as the reference point for all subjects in this long-term extension (LTE) study report to analyze all changes from baseline-related endpoints, unless otherwise specified. Efficacy and safety data were summarized using descriptive statistics. All analyses were pre-specified and conducted in line with those performed in the EDELWEISS 3 study to allow comparability.

There were no statistical comparisons between treatment groups in this extension study. Methods for examining efficacy endpoints in this LTE study were similar to those used in the main study EDELWEISS 3 (Donnez *et al.*, 2024). Primary endpoints included proportions of dysmenorrhea and NMPP responders at week 52 (end of treatment). As only the 200 mg+ABT was proven to be statistically significant in terms of its primary endpoint in the EDELWEISS 3 study, any further testing for superiority of the 200 mg+ABT over the 75 mg group was considered unnecessary.

Other efficacy endpoints were derived in the same way as done in the main study and summarized by descriptive statistics for each treatment group and each timepoint. Several additional secondary endpoints were identified to explore the change from baseline in various outcomes like dysmenorrhea, NMPP, overall pelvic pain (NRS and VRS), dyschezia, dyspareunia, analgesic use and opioid analgesic use for endometriosis-associated pain, EHP-30 dimensions (pain, control and powerlessness, emotional well-being, social support, self-image dimensions, and the modular sexual relationship questionnaire), and the physician/subject surgery intention questionnaire (PSIQ/SSIQ) (patient reported). The EuroQoL 5-dimension 5-level (EQ-5D-5L) questionnaire and monthly patient global impression of change (PGIC) and severity (PGIS) questionnaires were also utilized.

## Results

### Patients

A total of 356 patients who completed the 6-month treatment period in the main study entered the extension study and were treated. Overall, three subjects met the discontinuation criterion at Month 6, thus 353 patients in total were included in the treatment extension analysis set (placebo/75 mg linzagolix [n = 57], placebo/200 mg linzagolix plus ABT [n = 57], 75 mg linzagolix [n = 118], and 200 mg linzagolix plus ABT [n = 121]). The full disposition of subjects in the EDELWEISS 6 study is shown in Supplementary Fig. S1. Demographics and baseline characteristics of the long-term extension population were consistent with those of the original randomized population and were similar across all main study groups (Table 1).

**Table 1.** Baseline characteristics (treatment extension analysis set).

	Placebo/LGX 75 mg (N = 57)	Placebo/LGX 200 mg + ABT (N = 57)	LGX 75 mg (N = 118)	LGX 200 mg + ABT (N = 121)	Total (N = 353)
**Age (years), mean (SD)**	34.2 (6.6)	35.0 (7.7)	35.0 (6.3)	34.9 (6.8)	34.8 (6.7)
**Race**					
White, n (%)	57 (100.0%)	56 (98.2%)	116 (98.3%)	119 (98.3%)	348 (98.6%)
Black or African American, n (%)	0	1 (1.8%)	1 (0.8%)	1 (0.8%)	3 (0.8%)
Asian, n (%)	0	0	1 (0.8%)	0	1 (0.3%)
Other, n (%)	0	0	0	1 (0.8%)	1 (0.3%)
**BMI (kg/m^2^), mean (SD)**	23.41 (4.04)	24.77 (5.06)	24.83 (5.55)	23.83 (4.59)	24.25 (4.94)
**Time since first seeking medical diagnosis/treatment (years), mean (SD)**	5.76 (5.20)	4.56 (4.22)	5.13 (4.49)	5.36 (3.99)	5.22 (4.40)
**Pain scores**					
Baseline overall pelvic pain VRS*, mean (SD)	1.84 (0.41)	1.93 (0.41)	1.90 (0.40)	1.90 (0.39)	1.89 (0.40)
Baseline non-menstrual pelvic pain VRS*, mean (SD)	1.70 (0.45)	1.82 (0.47)	1.76 (0.45)	1.77 (0.43)	1.76 (0.45)
Baseline dysmenorrhea VRS*, mean (SD)	2.27 (0.41)	2.30 (0.45)	2.27 (0.39)	2.28 (0.40)	2.28 (0.41)
Baseline overall pelvic pain NRS, mean (SD)	5.97 (1.53)	6.37 (1.60)	6.00 (1.62)	6.16 (1.53)	6.11 (1.57)
Baseline dyspareunia (VRS), mean (SD)	1.93 (0.87)	2.00 (0.83)	1.94 (0.87)	2.12 (0.84)	2.01 (0.86)
Baseline EHP-30 pain dimension, mean (SD)	50.56 (15.40)	52.92 (13.45)	51.83 (14.30)	52.08 (15.00)	51.88 (14.55)
Baseline analgesic use on bleeding days* (pill count/day), mean (SD)	1.80 (1.49)	1.36 (1.17)	1.96 (1.83)	2.11 (1.79)	1.89 (1.68)
Baseline analgesic use on non-bleeding days* (pill count/day), mean (SD)	0.85 (1.03)	0.60 (0.74)	1.05 (1.27)	1.11 (1.12)	0.97 (1.12)
**Baseline dyschezia NRS, mean (SD)**	4.38 (2.22)	4.57 (2.76)	3.78 (2.70)	3.94 (2.75)	4.06 (2.66)
**Baseline lipid levels**					
LDL cholesterol (mg/dl), mean (SD)	109.9 (33.1)	116.7 (32.6)	115.4 (33.6)	117.0 (31.5)	115.3 (32.6)
Triglycerides (mg/dl), mean (SD)	88.8 (39.4)	93.0 (51.5)	97.2 (54.1)	91.0 (42.2)	93.0 (47.5)
**Baseline BMD**					
Femoral neck (g/cm^2^), mean (SD)	0.997 (0.120)	1.002 (0.148)	1.010 (0.140)	0.986 (0.143)	0.999 (0.139)
Lumbar spine (g/cm^2^), mean (SD)	1.224 (0.136)	1.194 (0.140)	1.210 (0.153)	1.180 (0.129)	1.199 (0.141)
Total hip (g/cm^2^), mean (SD)	1.034 (0.107)	1.029 (0.121)	1.043 (0.125)	1.021 (0.125)	1.032 (0.121)
Average duration of menstrual cycle* (days)	27.51 (3.23)	27.88 (3.41)	28.44 (3.00)	27.97 (3.04)	28.04 (3.13)
Average number of days with uterine bleeding* (days)	5.89 (1.58)	6.31 (2.12)	6.88 (2.59)	6.76 (2.15)	6.58 (2.25)

* Based on the two selected screening menstrual cycles.

ABT, add-back therapy; BMD, bone mineral density; EHP-30, endometriosis health profile-30, LDL: low-density lipoprotein; NRS, numeric rating scale; VRS, verbal rating scale.

### E2 levels

**Figure 1.**
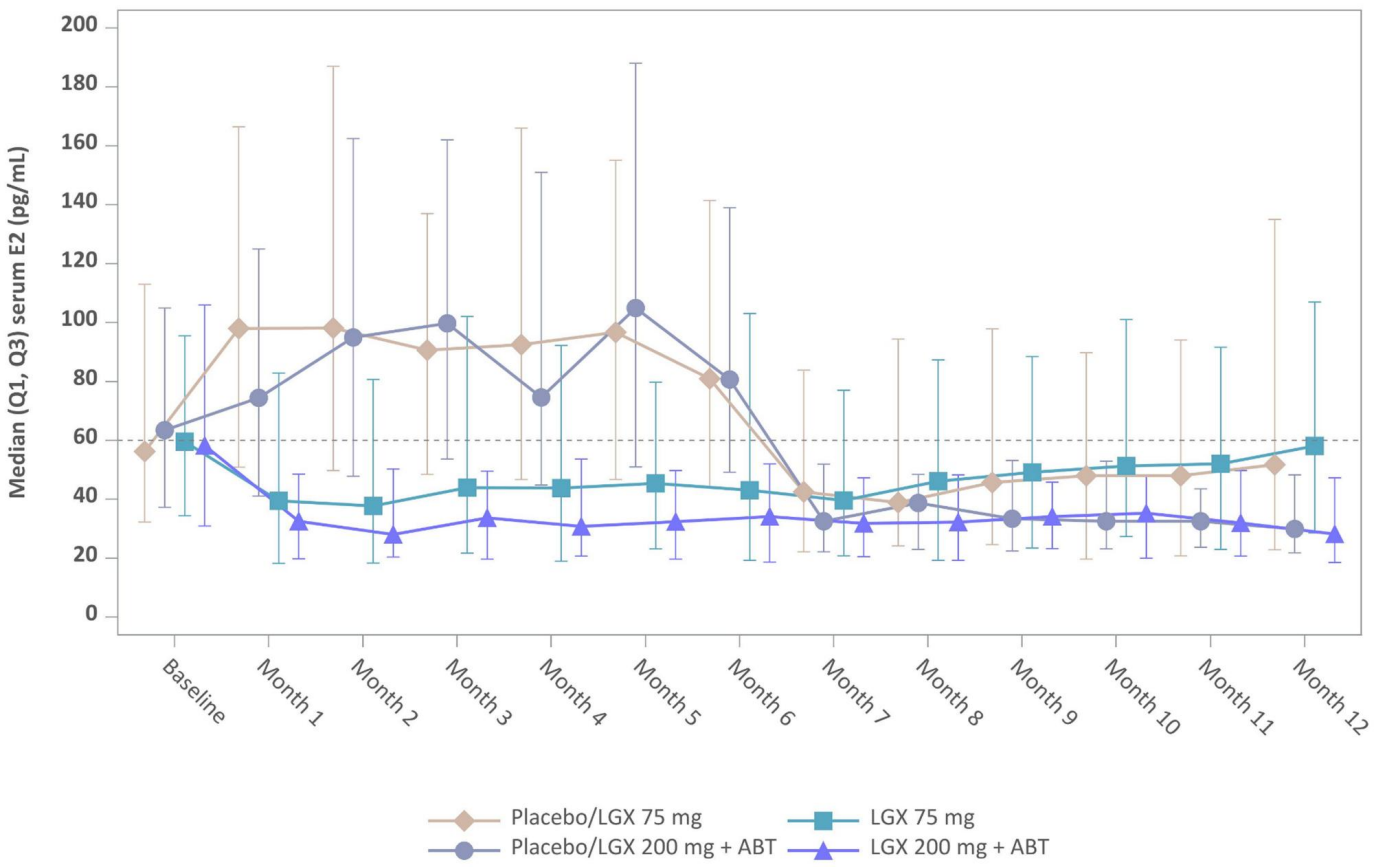
**Median estradiol levels at baseline during early follicular phase and under treatment**. Patients under placebo during the first 6 months (Edelweiss 3) were assigned to either LGX 75 mg or 200 mg + ABT in EDELWEISS 6 (Placebo/LGX 75 mg and Placebo/LGX 200mg + ABT groups, respectively). The other patients continued the treatment they were on. Error bars represent interquartile range. ABT, add-back therapy; E2, estradiol; LGX, linzagolix.

E2 levels were maintained within a therapeutic range (between 20 and 60 pg/ml), similar to values occurring naturally during the early follicular phase, in both patients given 75 mg linzagolix daily and those given 200 mg linzagolix with ABT daily (Fig. 1). In the linzagolix groups, there was a trend toward decreasing E2 levels from baseline to Month 12, with median E2 levels ranging between 39.50 and 58.10 pg/ml in the 75 mg linzagolix group, and 28.05 and 35.30 pg/ml in the 200 mg+ABT group. Most subjects in linzagolix groups had an E2 level <60 pg/ml throughout the treatment period, with a higher proportion of subjects in the 200 mg+ABT group. In the 75 mg group, E2 levels were suppressed to <20 pg/ml in ∼15–27% of subjects, while around 30–43% maintained E2 values ≥20 and <60 pg/ml throughout the 12-month treatment period. In the 200 mg+ABT group, 17–28% of subjects maintained an E2 level of <20 pg/ml, while 56–64% remained in the ≥20 and <60 pg/ml range throughout the treatment period.

Median E2 levels in the treatment-free ExFU period ranged from 93.85 to 98.90 pg/ml in the 75 mg linzagolix group and from 58.35 to 62.30 pg/ml in the 200 mg+ABT linzagolix group. The majority of subjects in the 75 mg group showed E2 levels of ≥60 pg/ml at Months 1 and 3 ExFU. In the 200 mg+ABT linzagolix group, 49.0% of subjects had E2 levels of ≥ 60 pg/ml at Month 1 ExFU, which increased to 52.8% by Month 3 ExFU. Menses resumed after cessation of therapy in both linzagolix groups; the estimated time to recovery of menses was 4–5 weeks due to the rapid recovery of ovarian function (assessed by sex steroid hormone blood tests).

### Efficacy

#### Co-primary endpoints

**Figure 2.**
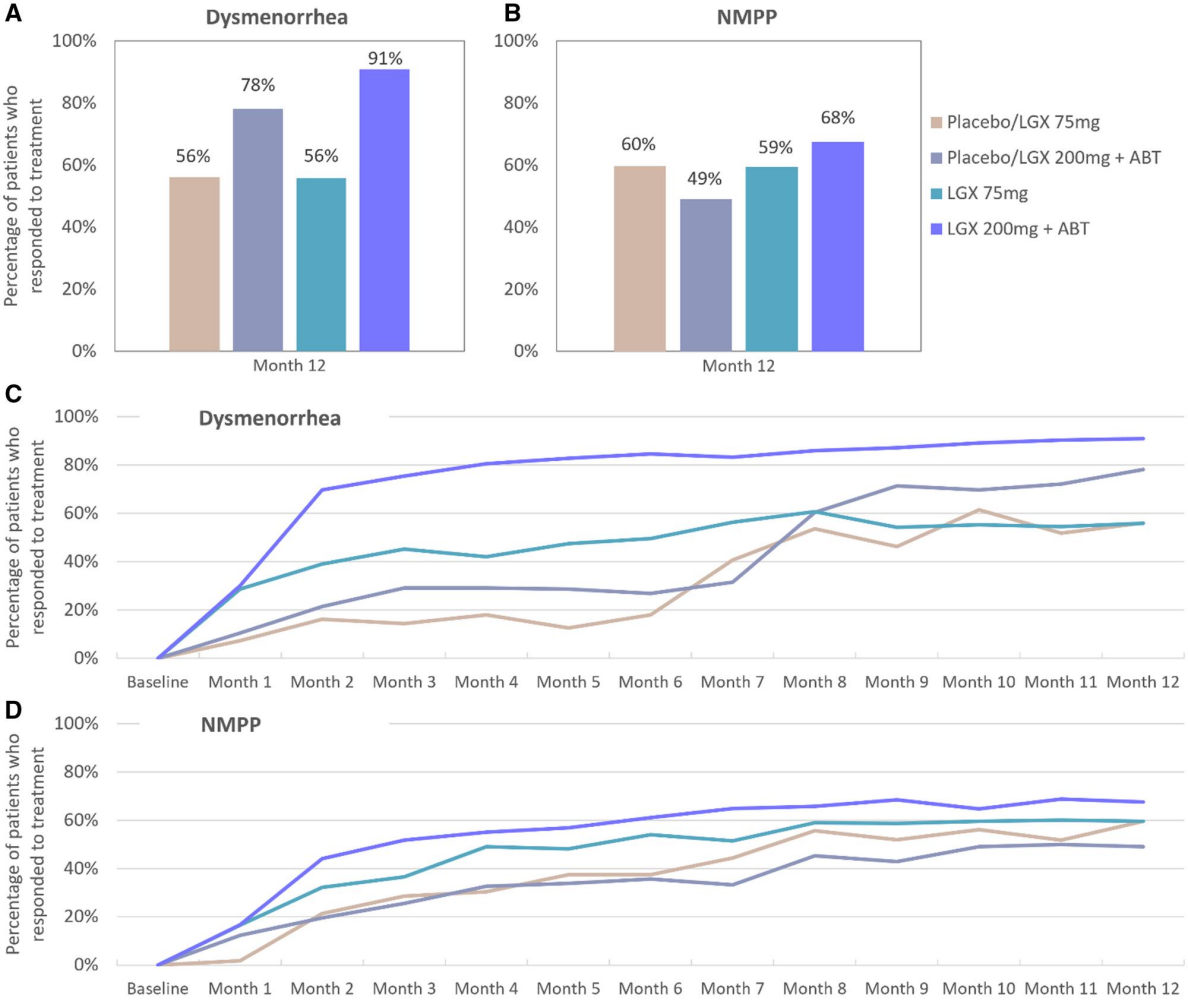
**Responder rates for reduction in dysmenorrhea and non-menstrual pelvic pain for patients under treatment**. Women were treated with placebo/LGX 75 mg, placebo/LGX 200 mg + ABT, LGX 75 mg or LGX 200 mg + ABT. (**A**) Proportion of patients who had a reduction in dysmenorrhea and (**B**) non-menstrual pelvic pain reduction at Month 12 and (**C**) changes in responder rates for dysmenorrhea and (**D**) non-menstrual pelvic pain from baseline to Month 12. Responders were defined as women with clinically meaningful reduction in pain and stable or decreased analgesic use. The responder thresholds for a clinically meaningful reduction in monthly pain scores were −1.1 and −0.8 for dysmenorrhea and non-menstrual pelvic pain, respectively, assessed on the verbal rating scale. ABT, add-back therapy; LGX, linzagolix; NMPP, non-menstrual pelvic pain.

At Month 12, the proportion of subjects with a reduction of 1.10 (Month 3 meaningful change threshold [MCT] = −1.10) or more in dysmenorrhea score (VRS) and stable or decreased use of analgesics was 55.9% in the 75 mg linzagolix group and 91.0% in the 200 mg+ABT linzagolix group (Fig. 2A). From Month 6, rates of women with a reduction for dysmenorrhea and stable or decreased use of analgesics in the 75 mg group rose further until Month 8, then dropped slightly by Month 9, remaining stable until Month 12. In the 200 mg+ABT linzagolix group, the percentage of subjects with reduced dysmenorrhea and stable or decreased use of analgesics continued to rise steadily from Month 6 until the end of treatment at Month 12 (Fig. 2C).

At Month 12, the proportion of women with a reduction of 0.80 (Month 3 MCT = −0.80) or more in NMPP (VRS) and stable or decreased use of analgesics was 59.5% in the 75 mg linzagolix group and 67.6% in the 200 mg+ABT linzagolix group (Fig. 2B). From Month 6, the percentage of subjects with a reduction in NMPP and stable or decreased use of analgesics in the 75 mg linzagolix group fell slightly at Month 7, then increased by Month 8, remaining stable until Month 12. In the 200 mg+ABT linzagolix group, rates of subjects with a reduction in NMPP and stable or decreased use of analgesics remained relatively steady from Month 6 until the end of treatment at Month 12 (Fig. 2D).

#### Secondary endpoints

**Figure 3.**
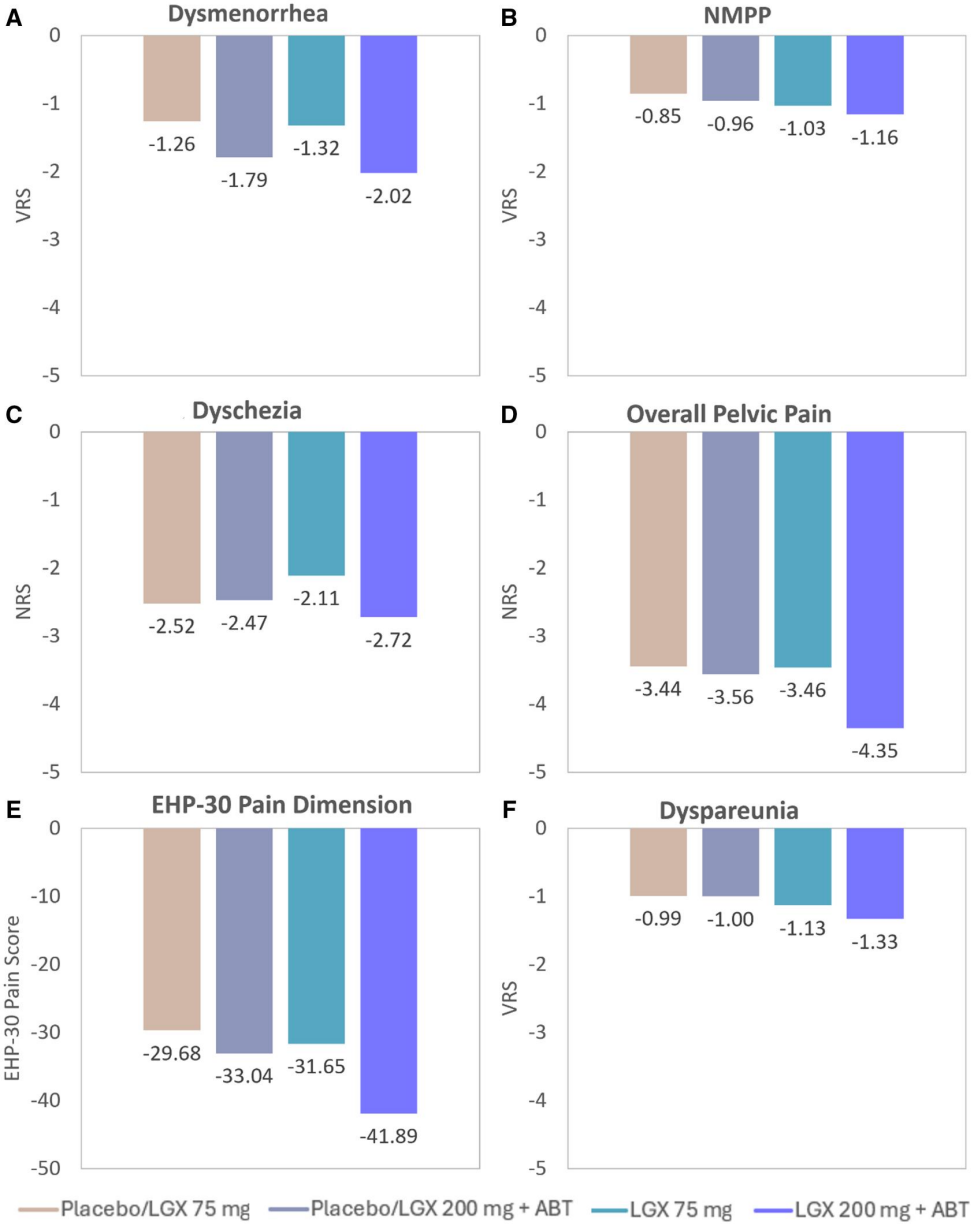
**Changes from baseline to Month 12 in pain measures for patients under treatment**. Women were treated with placebo/LGX 75mg, placebo/LGX 200mg + ABT, LGX 75mg, or LGX 200mg + ABT. (**A**) Dysmenorrhea (measured on a VRS), (**B**) Non-menstrual pelvic pain (measured on a VRS), (**C**) Dyschezia (measured on an NRS), (**D**) Overall pelvic pain (measured on an NRS), (**E**) Interference of pain with daily activities (pain dimension of EHP-30), and (**F**) Dyspareunia (measured on a VRS). ABT, add-back therapy; EHP-30, endometriosis health profile-30; LGX, linzagolix; NRS, numeric rating scale; VRS, verbal rating scale.

Steady improvements in dysmenorrhea were observed in both linzagolix groups throughout the 12-month treatment period, with greater improvement in the 200 mg+ABT linzagolix group. Mean change from baseline for dysmenorrhea (VRS) at Month 12 was −1.32 (vs −1.08 at Month 6) and −2.02 (vs −1.98 at Month 6) in the 75 mg linzagolix and 200 mg+ABT linzagolix groups, respectively (Fig. 3A). As already mentioned for the primary endpoint, upon end of treatment at Month 12, the proportion of subjects with reduced dysmenorrhea and stable or decreased use of analgesics fell in both linzagolix groups. Rates were 55.9% in the 75 mg linzagolix group and 91.0% in the 200 mg+ABT linzagolix group. At Month 6 after treatment cessation, the proportion of dysmenorrhea responders (VRS) with stable or decreased use of analgesics was 40.9% in the 75 mg linzagolix group and 54.3% in the 200 mg+ABT linzagolix group (Month 3 MCT analysis).

Marked improvements in NMPP were also observed in both linzagolix groups throughout the treatment period, again showing greater improvement with 200 mg+ABT linzagolix. Mean change from baseline in NMPP (VRS) at Month 12 was −1.03 (vs −0.86 at Month 6) with 75 mg linzagolix and −1.16 (vs −1.00 at Month 6) with the 200 mg+ABT linzagolix (Fig. 3B). Following the end of treatment at Month 12, rates of women with a reduction in NMPP and stable or decreased use of analgesics remained relatively steady in both linzagolix groups. The percentage was 59.5% in the 75 mg linzagolix group and 67.6% in the 200 mg+ABT linzagolix group. At Month 6 after treatment cessation, rates of NMPP responders were 55.7% with 75 mg linzagolix and 67.0% with 200 mg+ABT linzagolix (Month 3 MCT analysis).

A steady reduction in mean daily dyschezia scores was observed in both linzagolix groups from baseline to Month 12, with a more marked reduction in the 200 mg+ABT linzagolix group (Fig. 3C). Mean change from baseline in dyschezia at Month 12 was −2.11 (vs −1.85 at Month 6) in the 75 mg linzagolix group and −2.72 (vs −2.20 at Month 6) in the 200 mg+ABT linzagolix group. Mean daily dyschezia scores rose slightly after the end of therapy, showing a mean change from baseline 6 months after the end of treatment of −1.94 in the 75 mg linzagolix group and −2.16 in the 200 mg+ABT linzagolix group.

Marked improvements in mean overall pelvic pain scores (NRS) were observed in both linzagolix groups from baseline to Month 12, with the greatest improvement in the 200 mg+ABT linzagolix group. Mean change from baseline for overall pelvic pain scores at Month 12 was −3.46 (vs −2.92 at Month 6) in the 75 mg linzagolix group and −4.35 (vs −3.76 at Month 6) in the 200 mg+ABT linzagolix group (Fig. 3D). After the end of treatment, an increase in mean overall pelvic pain scores was noted in both linzagolix groups, although they did remain below scores seen at baseline, with −0.87 and −1.12 in the 75 mg linzagolix group and the 200 mg+ABT group, respectively.

Improvements in interference of pain with the ability to perform daily activities were also noted, with mean change from baseline in EHP-30 pain dimension scores at Month 12 of −31.65 (vs −28.13 at Month 6) with 75 mg linzagolix and −41.89 (vs −37.70 at Month 6) with 200 mg+ABT linzagolix (Fig. 3E). Six months after the end of therapy, mean change from baseline was −26.21 in the 75 mg linzagolix group and −34.38 in the 200 mg+ABT linzagolix group.

Mean daily dyspareunia scores were maintained in both linzagolix groups from Month 6 until the end of treatment at Month 12. Mean change from baseline for dyspareunia at Month 12 was −1.13 (vs −1.18 at Month 6) in the 75 mg linzagolix group and −1.33 (vs −1.16 at Month 6) in the 200 mg+ABT linzagolix group (Fig. 3F). Mean daily dyspareunia scores also remained relatively stable throughout the follow-up period. Mean change from baseline in dyspareunia scores at Month 6 post-treatment was −0.97 with 75 mg linzagolix and −1.11 with 200 mg+ABT linzagolix.

Similar steady decreases were encountered in the number of days of avoiding sexual intercourse because of anticipation of pain in both linzagolix groups from baseline to Month 12, showing mean changes of −5.10 and −6.79 in the 75 mg and 200 mg+ABT linzagolix groups, respectively.

Over the extension treatment period, the percentage of subjects not using analgesics for endometriosis-associated pain increased considerably in both linzagolix groups. At Month 12, rates of women not using analgesics for EAP was 45.2% (vs 34.8% at Month 6) in the 75 mg linzagolix group and 56.1% (vs 46.2% at Month 6) in the 200 mg+ABT linzagolix group. More than 95% of subjects in each of the linzagolix groups reported no opiate use for endometriosis-associated pain at Month 12.

**Figure 4.**
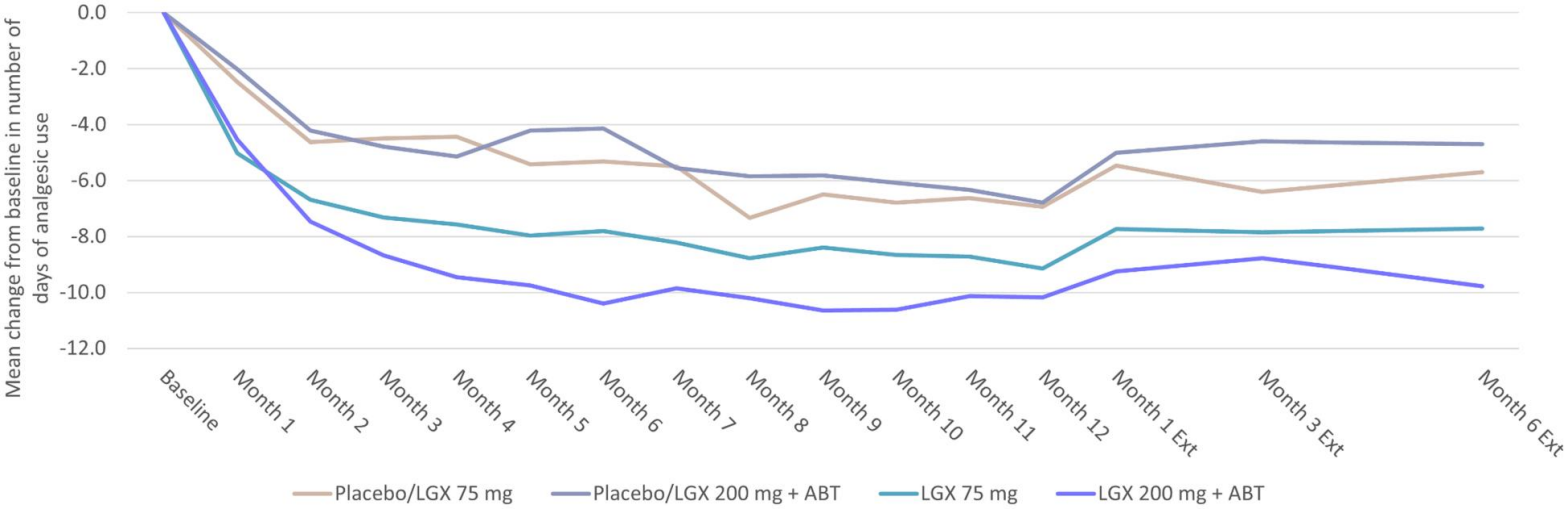
**Change from baseline to Month 12 (end of treatment) and Month 6 extension follow-up in mean number of days with any analgesics use for endometriosis-associated pain**. Women were treated with placebo/LGX 75 mg, placebo/LGX 200 mg + ABT, LGX 75 mg, or LGX 200 mg + ABT. ABT, add-back therapy; EXT, extension; LGX, linzagolix.

The number of days requiring analgesic use gradually fell in both linzagolix groups by the end of the treatment period at Month 12, with an estimated change from baseline at of −9.25 in the 75 mg linzagolix group and −10.23 in the 200 mg+ABT linzagolix group. The number of days with any analgesic use slightly increased during the 6-month treatment-free follow-up period, with an estimated mean change from baseline at Month 6 post-cessation of therapy of −7.72 in the 75 mg linzagolix group and −9.78 in the 200 mg+ABT linzagolix group (Fig. 4).

Quality of life was measured using the EHP-30 questionnaire. At Month 12, considerable improvements (total score reductions) were observed in all five dimensions in both linzagolix groups, with the most marked being in the 200 mg+ABT linzagolix group (except in terms of emotional well-being, where similar improvements were observed throughout).

Improvements in EHP-30 dimensions are detailed below.

*EHP-30 pain*: Mean change from baseline in EHP-30 pain dimension scores at Month 12 was −31.65 (vs −28.13 at Month 6) with 75 mg linzagolix and −41.89 (vs −37.70 at Month 6) with 200 mg+ABT linzagolix. Mean change from baseline after 6 months of treatment cessation was −26.21 in the 75 mg linzagolix group and −34.38 in the 200 mg+ABT linzagolix group.

*EHP-30 control and powerlessness*: Mean change from baseline in EHP-30 control and powerlessness dimension scores at Month 12 was −34.98 (vs −29.45 at Month 6) in the 75 mg linzagolix group and −48.28 (vs −41.56 at Month 6) in the 200 mg+ABT linzagolix group. Six months after the end of therapy, mean change from baseline was −29.38 with 75 mg linzagolix and −39.93 with 200 mg+ABT linzagolix.

*EHP-30 emotional well-being*: Mean change from baseline in EHP-30 emotional well-being scores at Month 12 was −24.41 (vs −21.05 at Month 6) in the 75 mg linzagolix group and −27.74 (vs −24.50 at Month 6) in the 200 mg+ABT linzagolix group. Six months after the end of therapy, mean change from baseline was −21.47 with 75 mg linzagolix and −24.61 with 200 mg+ABT linzagolix. EHP-30 social support: mean change from baseline in EHP-30 social support scores at Month 12 was −23.64 (vs −19.50 at Month 6) in the 75 mg linzagolix group and −30.90 (vs −26.83 at Month 6) in the 200 mg+ABT linzagolix group. Six months after the end of therapy, mean change from baseline was −21.81 with 75 mg linzagolix and −26.97 with 200 mg+ABT linzagolix.

*EHP-30 self-image*: Mean change from baseline in EHP-30 self-image dimension scores at Month 12 was −19.65 (vs −17.10 at Month 6) in the 75 mg linzagolix group and −25.15 (vs −22.34 at Month 6) in the 200 mg+ABT linzagolix group. Six months after the end of therapy, mean change from baseline was −16.41 with 75 mg linzagolix and −21.84 with 200 mg+ABT linzagolix.

A considerably reduced likelihood of surgery (measured by the PSIQ and SSIQ questionnaire) was observed at Month 12 (compared to Month 6).

Both linzagolix groups showed a gradual increase in EQ-5D-5L index values and visual analogue scale (VAS) scores from baseline to Month 12, with a more pronounced increase in the 200 mg+ABT linzagolix group. The mean change from baseline in EQ-5D-5L index values at Month 12 was 0.14 (vs 0.12 at Month 6) with 75 mg linzagolix and 0.17 (vs 0.14 at Month 6) with 200 mg+ABT linzagolix. At Month 6 ExFU, the mean change from baseline was 0.12 in the LGX 75 mg group and 0.16 in the LGX 200 mg+ABT group. Mean change from baseline in EQ-5D-5L VAS scores at Month 12 was 13.5 (vs 9.4 at Month 6) and 18.0 (vs 17.5 at Month 6), respectively. Six months after the end of therapy, mean change from baseline in EQ 5D-5L VAS scores was 11.9 in the 75 mg linzagolix group and 20.3 in the 200 mg+ABT linzagolix group.

Subject-perceived impression of change, as measured by responses to the PGIC, and severity, as gauged by responses to the PGIS, also showed improvements over the 12-month treatment period. A high proportion of women in both linzagolix groups considered their endometriosis symptoms at Month 12 to be either very much improved or much improved: 65.4% (vs 53.0% at Month 6) in the 75 mg linzagolix group and 86.5% (vs 82.9% at Month 6) in the 200 mg+ABT linzagolix group.

At Month 12, 77.6% and 88.1% of subjects in the LGX 75 mg and LGX 200 mg+ABT groups, respectively, had an improvement ≥1 in the PGIS score, and 34.6% and 48.6% of subjects in the LGX 75 mg and LGX 200 mg+ABT groups, respectively, had an improvement ≥2 in the PGIS score.

Mean baseline numbers of days with uterine bleeding (including spotting) were comparable between the LGX groups (75 mg LGX: 6.88 days; 200 mg+ABT LGX: 6.76 days). These durations steadily declined with both 75 mg LGX and 200 mg+ABT LGX until the end of the treatment period at 12 months, with a more pronounced decrease in the 200 mg+ABT group LGX. The mean change from baseline in the number of days with uterine bleeding (including spotting) at Month 12 was −2.62 (vs −2.29 at Month 6) and −5.26 (vs −5.03 at Month 6) for the 75 mg LGX and 200 mg+ABT LGX groups, respectively.

### Safety

Overall, linzagolix appeared to be well tolerated in the extension study (Table 2).

**Table 2.** Treatment-emergent adverse events up to Month 12 (extension safety analysis set).

	Placebo/LGX 75 mg (N = 58)	Placebo/LGX 200 mg + ABT (N = 57)	LGX 75 mg (N = 119)	LGX 200 mg + ABT (N = 122)	Total (N = 356)
**Number (%) of subjects with:**					
Any TEAE	37 (63.8)	39 (68.4)	75 (63.0)	83 (68.0)	234 (65.7)
Severe TEAE	1 (1.7)	3 (5.3)	3 (2.5)	2 (1.6)	9 (2.5)
TEAE related to Linzagolix	19 (32.8)	23 (40.4)	43 (36.1)	53 (43.4)	138 (38.8)
TEAE related to add-back therapy	17 (29.3)	16 (28.1)	31 (26.1)	43 (35.2)	107 (30.1)
Non-serious TEAE	37 (63.8)	39 (68.4)	75 (63.0)	83 (68.0)	234 (65.7)
Serious TEAE	0 (0.0)	1 (1.8)	4 (3.4)	0 (0.0)	5 (1.4)
Serious TEAE related to Linzagolix	0 (0.0)	0 (0.0)	0 (0.0)	0 (0.0)	0 (0.0)
Serious TEAE related to add-back therapy	0 (0.0)	0 (0.0)	1 (0.8)	0 (0.0)	1 (0.3)
TEAE leading to permanent discontinuation of treatment	3 (5.2)	2 (3.5)	4 (3.4)	2 (1.6)	11 (3.1)
Fatal TEAE	0 (0.0)	0 (0.0)	0 (0.0)	0 (0.0)	0 (0.0)
**TEAEs reported by ≥5% of subjects in any group** ^a^, n (%):					
Headache	4 (6.9)	11 (19.3)	12 (10.1)	15 (12.3)	42 (11.8)
Hot flush	5 (8.6)	1 (1.8)	13 (10.9)	12 (9.8)	31 (8.7)
COVID-19	4 (6.9)	3 (5.3)	10 (8.4)	10 (8.2)	27 (7.6)
Anaemia	7 (12.1)	5 (8.8)	10 (8.4)	3 (2.5)	25 (7.0)
Fatigue	2 (3.4)	2 (3.5)	9 (7.6)	9 (7.4)	22 (6.2)
Nausea	3 (5.2)	5 (8.8)	9 (7.6)	4 (3.3)	21 (5.9)
Mood swings	2 (3.4)	3 (5.3)	7 (5.9)	7 (5.7)	19 (5.3)
Vaginal haemorrhage	1 (1.7)	3 (5.3)	6 (5.0)	6 (4.9)	16 (4.5)
Bone density decreased	3 (5.2)	2 (3.5)	6 (5.0)	4 (3.3)	15 (4.2)
Diarrhoea	1 (1 .7)	5 (8.8)	4 (3.4)	5 (4.1)	15 (4.2)
Arthralgia	1 (1.7)	1 (1.8)	8 (6.7)	4 (3.3)	14 (3.9)
Nasopharyngitis	3 (5.2)	1 (1.8)	3 (2.5)	5 (4.1)	12 (3.4)
Vaginal infection	1 (1.7)	1 (1.8)	2 (1.7)	7 (5.7)	11 (3.1)
Vulvovaginal mycotic infection	3 (5.2)	3 (5.3)	2 (1.7)	3 (2.5)	11 (3.1)
Acne	3 (5.2)	2 (3.5)	1 (0.8)	4 (3.3)	10 (2.8)
Breast pain	3 (5.2)	2 (3.5)	3 (2.5)	2 (1.6)	10 (2.8)
Flatulence	1 (1.7)	0	6 (5.0)	3 (2.5)	10 (2.8)

a Events were sorted in decreasing order of frequency in the total column. In case of equal frequency, alphabetic order was used.

ABT, add-back therapy; LGX, linzagolix; TEAE, treatment-emergent adverse event.

More than 40% of subjects reported TEAEs between Month 6 and the end of treatment at Month 12 (Supplementary Table S1). The percentage of women reporting one or more TEAEs was comparable between the 75 mg linzagolix group (44.5%) and the 200 mg+ABT linzagolix group (40.2%). Most TEAEs (∼99% overall) were mild or moderate. The most commonly reported TEAEs from Month 6 to Month 12 were COVID-19 (4.2%), headaches (3.9%), and hot flushes (3.7%).

In the treatment-free follow-up period, only AEs occurring within 30 days after end of treatment were considered as treatment emergent. Of the 329 subjects included in the extension safety analysis set (ESAF), 32 subjects (9.7%) reported TEAEs (Supplementary Table S2). Rates of subjects experiencing one or more TEAEs were similar between linzagolix groups (75 mg: 9.8%; 200 mg+ABT: 9.7%).

Over the entire EDELWEISS 6 study, no TEAEs leading to deaths were reported in this study. The discontinuation rate due to TEAEs was 2.5% in the 75 mg linzagolix group and 1.6% in the 200 mg+ABT linzagolix group. There were no suicide-related TEAEs emerging from the study. Increases in lipid parameters were observed in both linzagolix groups at Month 12. Mean cholesterol levels increased from baseline by 4.84% and 3.81% in the 75 mg and 200 mg+ABT linzagolix groups, respectively and decreased by around 3% and 0.46%, respectively, by Month 3 after the end of therapy. Mean low-density lipoprotein cholesterol levels increased from baseline to Month 12 by 8.99% and 5.30% in the 75 mg and 200 mg+ABT linzagolix groups, respectively, and decreased by a mean of 1.88% and 1.33%, respectively by the Month 3 follow-up visit. Mean triglyceride levels rose from baseline to Month 12 by 17.03% and 24.31% in the 75 mg and 200 mg+ABT linzagolix groups, respectively, and fell by a mean of 6.34% and 9.35%, respectively by the Month 3 ExFU visit. Clinically significant laboratory values were infrequent in all groups, including increases in liver enzymes. There were no patterns or trends in any other laboratory parameters in any treatment group.

Changes in BMD were minimal. At Month 12 and up to Month 6 after the end of therapy, most subjects registered either no change or increase, or a decrease of no more than 3% in BMD.

**Figure 5.**
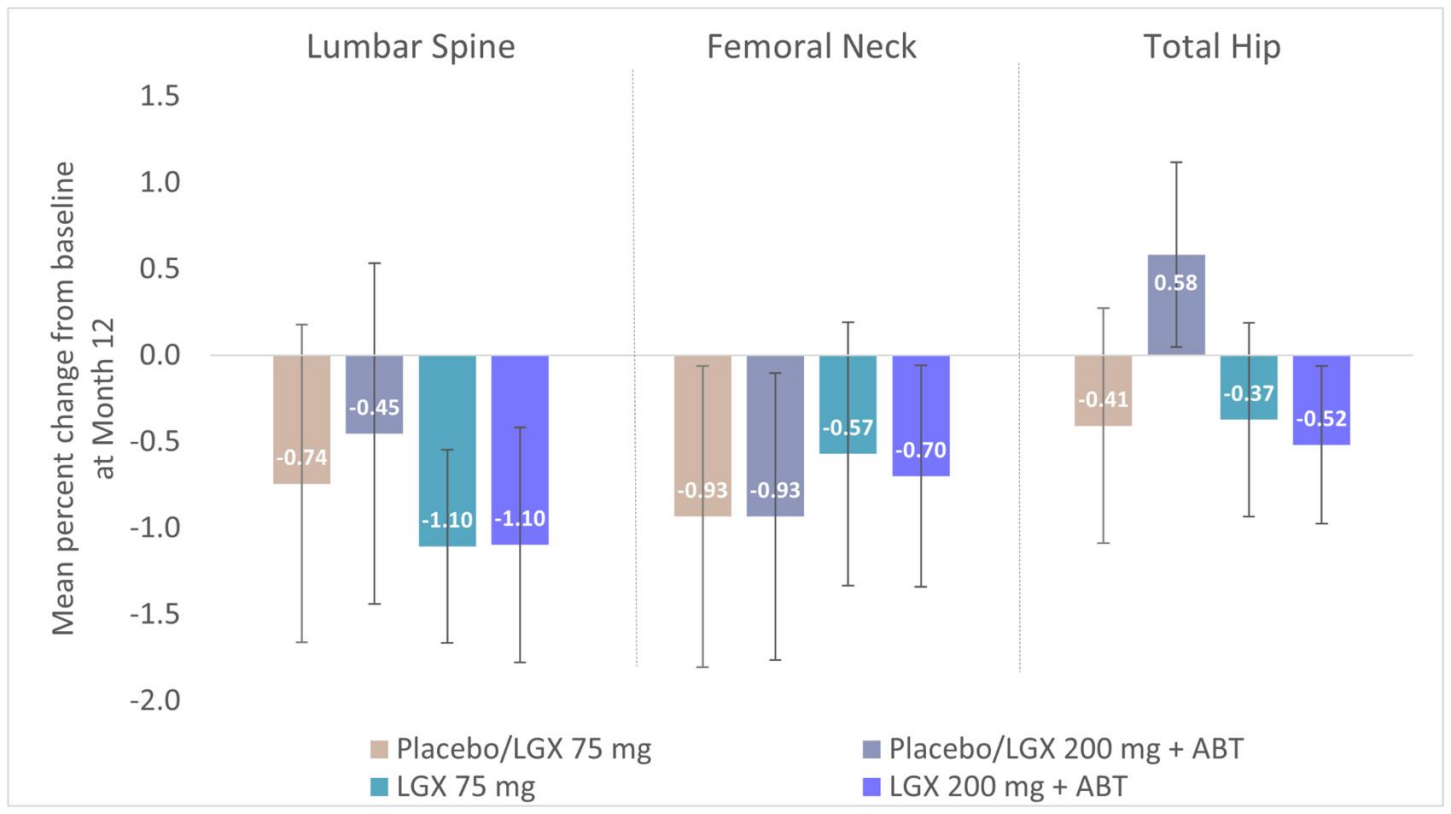
**Mean percent change from baseline in bone mineral density (BMD) at Month 12 for lumbar spine, femoral neck, and total hip**. Women were treated with placebo/LGX 75mg, placebo/LGX 200mg + ABT, LGX 75mg, or LGX 200mg + ABT. BMD was measured by DXA (dual energy X-ray absorptiometry). Error bars represent 95% CIs. ABT, add-back therapy; LGX, linzagolix.

The mean percentage change from baseline to Month 12 in the lumbar spine was −1.10% with both 75 mg linzagolix and 200 mg+ABT linzagolix. In the femoral neck, values were −0.57% in the 75 mg linzagolix group and −0.70% in the 200 mg+ABT linzagolix group. In the total hip, the mean percentage change from baseline was −0.37% with 75 mg linzagolix and −0.52% with 200 mg+ABT linzagolix (Fig. 5).

**Figure 6.**
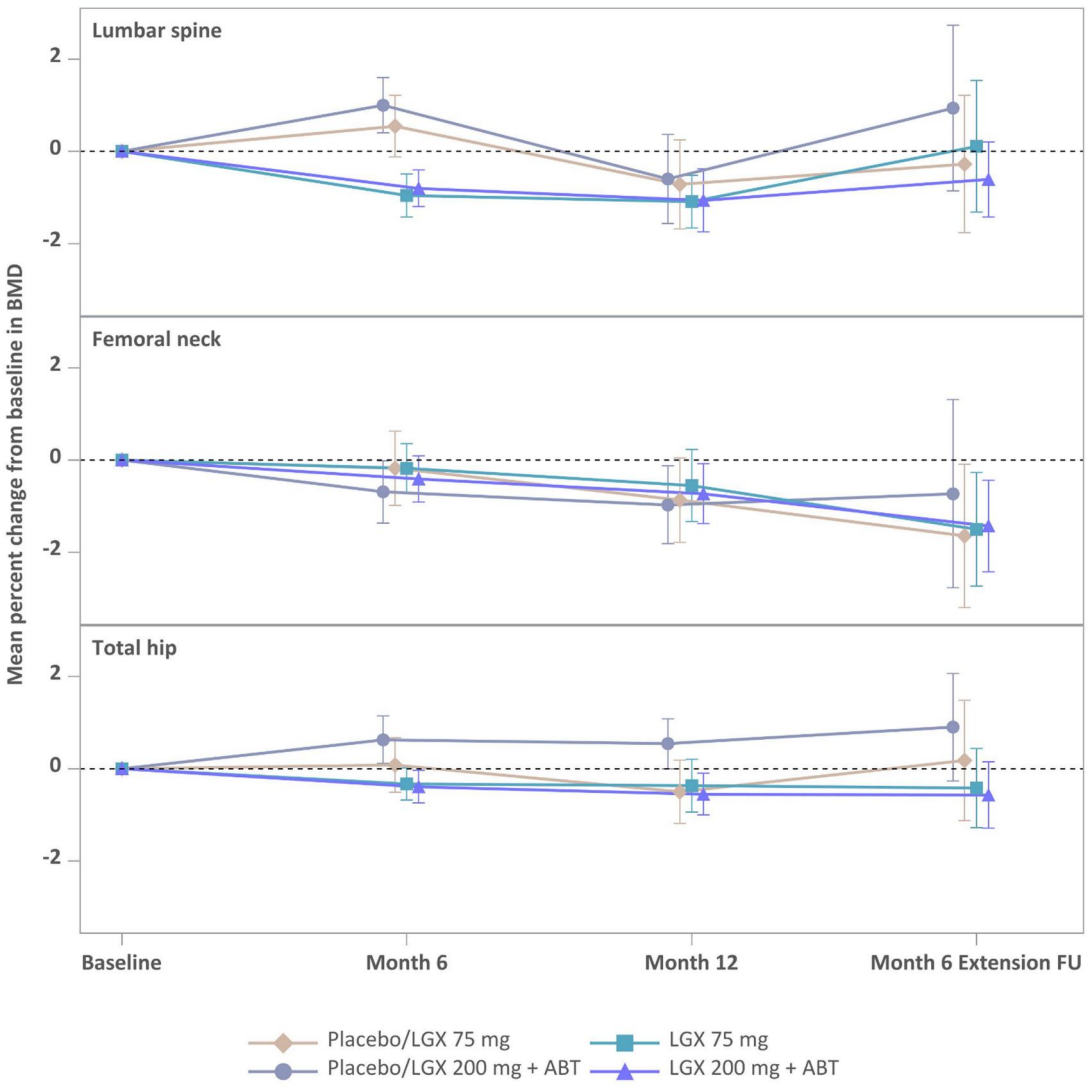
**Mean percent change from baseline to Month 6 extension follow-up in bone mineral density (BMD) for lumbar spine, femoral neck, and total hip**. Women were treated with placebo/LGX 75mg, placebo/LGX 200mg + ABT, LGX 75mg, or LGX 200mg + ABT. BMD was measured by DXA (dual energy X-ray absorptiometry). Error bars represent 95% CIs. ABT, add-back therapy; FU, follow-up; LGX, linzagolix.

The mean percentage change from baseline to Month 6 ExFU in the lumbar spine was 0.11% in the 75 mg linzagolix group and −0.61% in the 200 mg+ABT linzagolix group. In the femoral neck, ExFU values were −1.50% with 75 mg linzagolix and −1.43% with 200 mg+ABT linzagolix. The mean percentage change from baseline to Month 6 ExFU in the total hip was −0.42% in the 75 mg linzagolix group and −0.57% in the 200 mg+ABT linzagolix group (Fig. 6).

Decreases of more than 8% in BMD at the lumbar spine at Month 12 were reported in one patient in the 75 mg linzagolix group and in two in the 200 mg+ABT linzagolix group and for no patient 6 months after end of treatment. In the femoral neck, they were recorded in one patient in the Placebo/75 mg linzagolix group, in two in the 75 mg linzagolix group, and in one in the 200 mg+ABT linzagolix group at Month 12 and Month 6 ExFU. One patient in the 200 mg+ABT linzagolix group showed a decrease of more than 8% in BMD in the total hip at Month 12 and one patient showed the decrease at Month 6 ExFU.

Minimum Month 12 *Z*-scores did not drop below −2.0 in the lumbar spine, −1.4 in the femoral neck or −1.5 in the total hip. The minimum change from baseline was comparable across linzagolix groups in all bone sites. Small fluctuations in median *Z*-score values between baseline and Month 12 were observed in all groups, with no defined pattern.

There were no clinically meaningful changes from baseline in vital signs, physical examinations, electrocardiograms, gynecological examinations, breast examinations, endometrial biopsies histology or TVUS findings in any group. Overall, no new safety signals were raised in this extension study. All safety findings were in line with a previously described safety profile of linzagolix.

No pregnancies occurred during the EDELWEISS 6 extension study while under treatment.

## Discussion

Endometriosis is an estrogen-dependent chronic inflammatory disease that requires long-term management (Lousse *et al.*, 2012; Patel *et al.*, 2018; Donnez and Dolmans, 2021a,b; Taylor *et al.*, 2021; Horne and Missmer, 2022). While the first-line therapies are COCs or progestogens (Vercellini *et al.*, 2016, 2018a,b; Casper, 2017; Vannuccini *et al*., 2022), one-third of patients with endometriosis-related pain are not responsive due to progesterone resistance in their lesions (Flores *et al.*, 2018; Reis *et al.*, 2020; Donnez and Dolmans, 2021a; Bulun *et al.*, 2023). The second-line approach, the use of GnRH agonists without hormonal ABT, is limited to 6 months because they are associated with serious TEAEs like BMD loss and high rates of vasomotor effects (Donnez *et al.*, 2002, 2023, 2024; Olive, 2008; Donnez and Dolmans, 2021; Becker *et al.*, 2022).

There now exist new medical treatment options, as demonstrated by findings from clinical trials on three oral GnRH antagonists, whose advantages include oral administration, no flare-up effect, and rapid reversibility of a dose-dependent decrease in ovarian steroid secretion (Taylor *et al.*, 2017; Donnez and Dolmans, 2021b; Giudice *et al.*, 2022). One of the first papers on this reported 6-month outcomes of high-and low-dose elagolix monotherapy (Taylor *et al.*, 2017). A twice-daily dose of 200 mg elagolix yielded higher dysmenorrhea and NMPP responder rates than the lower once-daily dose (150 mg/daily). Responder rates for dysmenorrhea and NMPP were 75–78% and 67–69% at the higher elagolix dose (200 mg twice a day). At a dose of 150 mg daily, clinically meaningful responses were seen in 52% of women for dysmenorrhea and 67% for NMPP. Patients given the 200 mg dose of elagolix showed a greater decline in BMD, namely −3.6% and −3.9% at weeks 36 and 52, respectively (Surrey *et al.*, 2018). In an extension study on relugolix (Becker *et al.*, 2024), sustained improvements in endometriosis-associated pain were noted through 104 weeks among patients taking relugolix combination therapy. Responder rates at week 104 for dysmenorrhea and NMPP were 84.8% and 75.8%, respectively. After initial least square mean BMD loss of less than 1% at week 24, BMD plateaued by week 36 and was sustained for the duration of 104 weeks of treatment.

In our first paper, we evaluated two dose regimens for linzagolix: namely a once daily 200 mg dose in combination with hormonal ABT and a 75 mg dose without ABT, in women with moderate-to-severe endometriosis-related pain (Donnez *et al.*, 2024). In this phase 3 EDELWEISS 3 trial, dysmenorrhea and NMPP were significantly alleviated with 200 mg+ABT at 3 and 6 months compared to a placebo. The 75 mg dose missed the statistical significance for NMPP at Month 3 but could demonstrate statistical significance at Month 6. A dose of 75 mg linzagolix was chosen based on results from a phase 2A study (Donnez *et al.*, 2020). In the Edelweiss trials, the effect of 75 mg linzagolix was less pronounced than 200 mg linzagolix and became statistically significant at 6 and 12 months. Given the dose-dependent response, it would be interesting to see how the 100 mg dose used in the fibroid trials would perform in this context.

In the current study reporting data from the EDELWEISS 6 extension study, it was demonstrated that efficacy observed during the initial 24 weeks of treatment in the pivotal EDELWEISS 3 study (Donnez *et al.*, 2024) was maintained over time with continued therapy. Indeed, in a population of 353 women with moderate-to-severe pain associated with endometriosis, once-daily oral 200 mg+ABT linzagolix or 75 mg linzagolix alone provided sustained and clinically meaningful reductions in dysmenorrhea and NMPP for up to 12 months, achieving the co-primary endpoints.

The baseline characteristics and the outcomes at Month 6 of the Edelweiss 3 and 6 populations this comparison was previously made but did not demonstrate any major difference.

By the end of treatment at Month 12, proportions of subjects with reduced dysmenorrhea and stable or decreased use of analgesics were 55.9% in the 75 mg linzagolix group and 91% in the 200 mg+ABT linzagolix group. As expected, and by definition, a significant reduction in and often complete cessation of menses with treatment did, of course, eliminate dysmenorrhea. As this is often the greatest concern among patients with the disease, it is important to point out that this most bothersome symptom is significantly improved with treatment.

Rates of women showing reduced NMPP with steady or declining use of analgesics was 59.5% with 75 mg linzagolix and 67.6% with 200 mg+ABT linzagolix. As in the 24-week evaluation, higher responder rates were observed for dysmenorrhea in the 200 mg+ABT group than in the 75 mg group, with the higher dose curtailing the number of bleeding days more effectively. Uterine bleeding was not completely eliminated by treatment with 200 mg+ABT linzagolix, probably because the ABT itself caused some uterine bleeding/spotting, which explains why dysmenorrhea was not completely eliminated in this group either.

More women experienced a reduction in dysmenorrhea than in NMPP. Among women treated with 200 mg+ABT linzagolix, percentages of those with decreased dysmenorrhea (72.9%, 80.0%, and 91.0% at Months 3, 6, and 12, respectively) were higher than those with reduced NMPP (47.3%, 57.1%, and 62.6%). A similar observation was made by Taylor *et al.* (2017) and by Surrey *et al.* (2018), Giudice *et al.* (2022), and Becker *et al.* (2024) in women given elagolix or relugolix combination therapy. Indeed, mechanisms governing NMPP are multifactorial, involving a peritoneal inflammatory environment, pelvic adhesions, generation of myofascial trigger points, and central sensitization. These mechanisms are probably less responsive to lower levels of E2 than dysmenorrhea (Aredo *et al.*, 2017; Giudice *et al.*, 2022; Donnez *et al.*, 2024).

Clinically meaningful improvements were also observed in other crucial secondary endpoints, specifically dyschezia, dyspareunia, overall pelvic pain, and the capacity to perform normal daily activities, as gauged by the EHP-30 pain dimension scale. Efficacy for pain was indicated by decreased analgesic and opioid use.

All significant changes in primary and secondary endpoints were consistent with the rapid and differential suppression of serum E2 levels, which remained in the so-called optimal zone (20– 60 pg/ml) through most of the treatment. The incidence of serious and non-serious side effects was low, and similar across both linzagolix groups, suggesting good tolerability. There were no fatalities or suicide-related incidents in the study. No pregnancies were reported in subjects given 200 mg + ABT linzagolix, notably because it is known to induce full suppression of ovarian activity and anovulation (Donnez *et al.*, 2022) after at least 2 weeks of therapy. Changes in BMD were minimal, with no clinically meaningful modifications from Month 6 to Month 12. Mean percentage change from baseline to Month 12 in the lumbar spine was −1.10% with both 75 mg linzagolix and 200 mg+ABT linzagolix, and less than −0.70% in other skeletal sites investigated.

Finally, dose-dependent estradiol suppression and rapid reversibility of pain relief due to the short half-life are key issues for women with endometriosis, which means that clinicians need to select the optimal dose, be it 75 mg alone (currently not on the market) or 200 mg + ABT. Linzagolix may have an advantage in terms of patient compliance, as it can be administered in a single dose at any time during the day (Donnez *et al.*, 2024). The present study demonstrates that high doses of linzagolix with ABT are effective (like relugolix with ABT, Becker *et al.*, 2024), but also that low doses without ABT can significantly alleviate both dysmenorrhea and NMPP by 12 months of therapy. This opens up the prospect of treatment for women in whom COCs or progestogens are contraindicated, or for women who are reluctant to take them.

Efficacy for dysmenorrhea, NMPP, overall pelvic pain, dyschezia, and dyspareunia did decline somewhat after treatment cessation, but improvements compared to baseline were generally still observed up to 6 months post-treatment in both the 75 mg linzagolix and 200 mg+ABT linzagolix groups. The observation that dysmenorrhea and NMPP remain assuaged for several months after cessation of therapy allows us to consider the possibility of intermittent therapy.

The strength of the current extension study is that it was a multinational, multi-centered, RCT in patients with endometriosis-associated pain, looking to assess the persistence of linzagolix efficacy and safety observed at Month 6 and compare two different doses administered orally: 75 mg alone or 200 mg +ABT. Assessments of pain were based primarily on patient-reported outcomes recorded daily in eDiaries using a VRS for endometriosis-associated pain. Safety analyses included controlled evaluations of BMD loss at 12, 24, and 52 weeks.

This study also has limitations. Data from comparisons with estrogens and progestogens or progestogens alone would be beneficial to determine whether GnRH antagonists offer any advantages over the traditional first-line medications. Regarding endometriosis-related symptoms, we strongly endorse the use of first-line therapies (COCs or progestogens) (Vercellini *et al.*, 2018, 2019, 2025; Donnez and Dolmans, 2021a,b; Donnez *et al.*, 2023). However, if these treatments fail, GnRH antagonists can work through a different mechanism, namely estrogen deprivation, and may be effective even in subjects with progesterone-resistant endometriosis (Donnez and Dolmans, 2021a,b; Donnez *et al.*, 2023). Thus long-term therapy, designed to preferably inhibit ovulation or alternatively reduce estrogen production, is recommended for this chronic condition (Practice Committee of the American Society for Reproductive Medicine, 2014; Zondervan *et al.*, 2020; Becker *et al.*, 2022), yet the costs of any long-term medical treatments need to be carefully balanced (Donnez and Dolmans, 2021a,b). As stated by Vercellini *et al.* (2018a, b, 2019) and by ourselves (Donnez *et al.*, 2024), further studies should investigate the cost-effectiveness and additional benefits of GnRH antagonists in women who are poor responders to progestogen therapy because of the widely recognized phenomenon of progesterone resistance.

## Conclusion

For women with endometriosis, clinically meaningful improvements in dysmenorrhea, NMPP, dyschezia, dyspareunia, overall pelvic pain, daily activities, and use of analgesics and opioids observed at Month 6 were generally found to have increased by Month 12 with both 75 mg linzagolix and 200 mg+ABT linzagolix. Overall, the efficacy and safety findings from the current extension study enable clinicians to personalize the medical approach according to the patient’s symptoms and wishes.

## Supplementary Material

hoag030_Supplementary_Data

## Data Availability

Appropriately de-identified patient-level datasets and supporting documents may be shared following the attainment of applicable marketing approvals and consistent with criteria established by Theramex and/or industry best practices to maintain the privacy of study participants.
