# Supplementary material for: Linzagolix, with and without add-back therapy, in women with endometriosis-associated pain: results from EDELWEISS 6, a double-blind randomized extension and withdrawal study
Source: Hum Reprod Open. 2026 Apr 8;2026(2):hoag030. doi: 10.1093/hropen/hoag030 (PMC13135359; doi:10.1093/hropen/hoag030)
Supplement: hoag030_Supplementary_Data [file hoag030_supplementary_data.zip › HRO-25-0511-R3-SuppFigureS1_EO.docx]

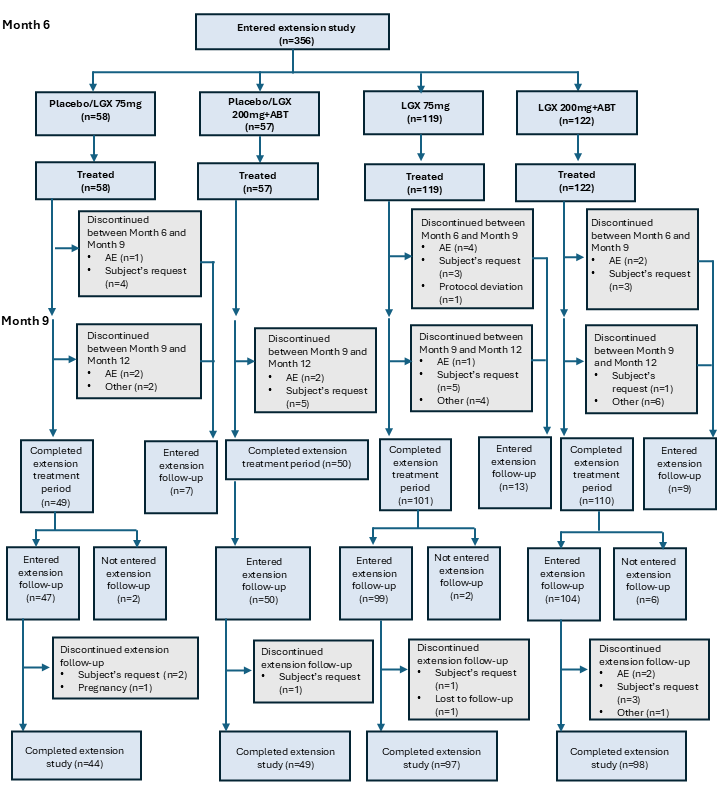


**Supplementary Figure S1. Subject disposition between start of EDELWEISS 6 and study completion at 6 months after end of treatment.** ABT, add-back therapy; AE, adverse event; LGX, linzagolix.
