# Supplementary material for: Linzagolix, with and without add-back therapy, in women with endometriosis-associated pain: results from EDELWEISS 6, a double-blind randomized extension and withdrawal study
Source: Hum Reprod Open. 2026 Apr 8;2026(2):hoag030. doi: 10.1093/hropen/hoag030 (PMC13135359; doi:10.1093/hropen/hoag030)
Supplement: hoag030_Supplementary_Data [file hoag030_supplementary_data.zip › HRO-25-0511-R3-SuppTablesS1andS2_EO.docx]

**Supplementary Table S1. Treatment-emergent adverse events from Month 6 to Month 12 (Extension Safety Analysis Set)**

|  | Placebo/ LGX 75 mg | Placebo/  LGX 200 mg + ABT | LGX 75 mg | LGX 200 mg + ABT | Total |
| --- | --- | --- | --- | --- | --- |
|  | (N=58) | (N=57) | (N=119) | (N=122) | (N=356) |
| Number (%) of subjects with: |  |  |  |  |  |
| Any TEAE | 27 (46.6) | 27 (47.4) | 53 (44.5) | 49 (40.2) | 156 (43.8) |
| Severe TEAE | 1 (1.7) | 2 (3.5) | 1 (0.8) | 1 (0.8) | 5 (1.4) |
| TEAE related to Linzagolix | 13 (22.4) | 14 (24.6) | 19 (16.0) | 23 (18.9) | 69 (19.4) |
| TEAE related to add-back therapy | 10 (17.2) | 8 (14.0) | 14 (11.8) | 13 (10.7) | 45 (12.6) |
| Non-serious TEAE | 27 (46.6) | 27 (47.4) | 53 (44.5) | 49 (40.2) | 156 (43.8) |
| Serious TEAE | 0 (0.0) | 1 (1.8) | 3 (2.5) | 0 (0.0) | 4 (1.1) |
| Serious TEAE related to Linzagolix | 0 (0.0) | 0 (0.0) | 0 (0.0) | 0 (0.0) | 0 (0.0) |
| Serious TEAE related to add-back therapy | 0 (0.0) | 0 (0.0) | 1 (0.8) | 0 (0.0) | 1 (0.3) |
| TEAE leading to permanent discontinuation of treatment | 2 (3.4) | 2 (3.5) | 3 (2.5) | 2 (1.6) | 9 (2.5) |
| Fatal TEAE | 0 (0.0) | 0 (0.0) | 0 (0.0) | 0 (0.0) | 0 (0.0) |
|  |  |  |  |  |  |
| TEAEs reported by ≥5% of subjects in any group^a^, n (%): |  |  |  |  |  |
| COVID-19 | 1 (1.7) | 2 (3.5) | 6 (5.0) | 6 (4.9) | 15 (4.2) |
| Headache | 3 (5.2) | 3 (5.3) | 5 (4.2) | 3 (2.5) | 14 (3.9) |
| Hot flush | 4 (6.9) | 0 | 4 (3.4) | 5 (4.1) | 13 (3.7) |
| Anaemia | 3 (5.2) | 2 (3.5) | 7 (5.9) | 0 | 12 (3.4) |
| Vaginal haemorrhage | 1 (1.7) | 3 (5.3) | 3 (2.5) | 3 (2.5) | 10 (2.8) |
| Vulvovaginal mycotic infection | 3 (5.2) | 3 (5.3) | 1 (0.8) | 2 (1.6) | 9 (2.5) |

ABT, add-back therapy; LGX, linzagolix; TEAE, treatment-emergent adverse event.

**Supplementary Table S2. Adverse events from Month 12 to Month 6 Extension follow-up (Follow-up Safety Analysis Set)**

|  | Placebo/ LGX 75 mg | Placebo/  LGX 200 mg + ABT | LGX 75 mg | LGX 200 mg + ABT | Total |
| --- | --- | --- | --- | --- | --- |
|  | (N=54) | (N=50) | (N=112) | (N=113) | (N=329) |
| Number of subjects (%) with: |  |  |  |  |  |
| Any AE | 17 (31.5) | 9 (18.0) | 31 (27.7) | 41 (36.3) | 98 (29.8) |
| Any TEAE | 7 (13.0) | 3 (6.0) | 11 (9.8) | 11 (9.7) | 32 (9.7) |
| Any post-treatment AE | 11 (20.4) | 6 (12.0) | 22 (19.6) | 35 (31.0) | 74 (22.5) |
| Severe TEAE | 0 (0.0) | 0 (0.0) | 0 (0.0) | 0 (0.0) | 0 (0.0) |
| TEAE related to Linzagolix | 1 (1.9) | 0 (0.0) | 2 (1.8) | 4 (3.5) | 7 (2.1) |
| TEAE related to add-back therapy | 0 (0.0) | 0 (0.0) | 0 (0.0) | 1 (0.9) | 1 (0.3) |
| Non-serious TEAE | 7 (13.0) | 3 (6.0) | 11 (9.8) | 11 (9.7) | 32 (9.7) |
| Serious TEAE | 0 (0.0) | 0 (0.0) | 0 (0.0) | 0 (0.0) | 0 (0.0) |
| Serious TEAE related to Linzagolix | 0 (0.0) | 0 (0.0) | 0 (0.0) | 0 (0.0) | 0 (0.0) |
| Serious TEAE related to add-back therapy | 0 (0.0) | 0 (0.0) | 0 (0.0) | 0 (0.0) | 0 (0.0) |
| Fatal TEAE | 0 (0.0) | 0 (0.0) | 0 (0.0) | 0 (0.0) | 0 (0.0) |

ABT, add-back therapy; LGX, linzagolix; TEAE, treatment-emergent adverse event
